# Consumption of non-antibacterial drugs may have negative impact on *Helicobacter pylori* colonization in the stomach

**DOI:** 10.1016/j.heliyon.2024.e27327

**Published:** 2024-03-04

**Authors:** Allah Nazar Atif, Atousa Hatefi, Asadullah Arven, Alireza Foroumadi, Sara Kadkhodaei, Alireza Sadjadi, Farideh Siavoshi

**Affiliations:** a Department of Microbiology, School of Biology, University College of Sciences, University of Tehran, Tehran, Iran; b Department of Biology, Faculty of Sciences, Nangarhar University, Jalalabad, Afghanistan; c Department of Biology, Faculty of Education, Daykundi University, Nilli, Afghanistan; d Department of Medicinal Chemistry, Faculty of Pharmacy and Drug Design & Development Research Center, The Institute of Pharmaceutical Sciences (TIPS), Tehran University of Medical Sciences, Tehran, Iran; e Digestive Disease Research Institute, Tehran University of Medical Sciences, Tehran, Iran

**Keywords:** *Helicobacter pylori*, Non-antibacterial drugs, Susceptibility, Minimum inhibitory concentration, Plasma concentration

## Abstract

**Background:**

Nineteen non-antibacterials were examined to show that their consumption for treatment of other diseases may inhibit *Helicobacter pylori*. Four antibiotics were used for comparison.

**Materials and methods:**

Agar dilution method was used to examine the susceptibility of 20 *H. pylori* isolates to 4 antibiotics; metronidazole (MTZ), clarithromycin (CLR), amoxicillin (AMX), tetracycline (TET) and 19 non-antibacterials; proton pump inhibitors (PPIs), H_2_-blockers, bismuth subsalicylate (BSS), antifungals, statins, acetaminophen (ACE), aspirin (ASA), B-vitamins (B-Vits; Vit B1, Vit B6 and Vit B_complex)_ and vitamin C (Vit C). Blood agar plates were prepared with different concentrations of drugs and spot-inoculated with bacterial suspensions. Plates were incubated at 37 °C under microaerobic conditions and examined after 3–5 days. The isolate #20 that was mucoid and resistant to 19 drugs, including MTZ and SMV was tested against combined MTZ (8 μg/mL) and SMV (100 μg/mL). Results were analyzed statistically.

**Results:**

Minimum inhibitory concentrations (MICs, μg/mL) of drugs and the frequency of susceptible *H. pylori* were determined as MTZ (8, 80%), CLR (2, 90%), AMX (1, 100%), TET (0.5, 70%), PPIs (8–128, 80%), H_2_-blockers (2000–8000, 75–80%), BSS (15, 85%), antifungals (64–256, 30–80%), statins (100–250, 35–90%), ACE (40, 75%), ASA (800, 75%), B-Vits (5000–20000, 80–100%) and Vit C (2048, 85%). Susceptibility of *H. pylori* isolates to 16 out of 19 non-antimicrobials (75–100%) was almost similar to those of antibiotics (70–100%) (*P*-value >0.05). The highest susceptibility rate (100%) belonged to Vit B1, Vit B6 and AMX. Out of 20 *H. pylori* isolates, 17 (85%) were susceptible to ≥13 non-antimicrobials and 3 (15%) were susceptible to < 13 (*P*-value <0.05). Mucoid *H. pylori* showed susceptibility to combination of MTZ and SMV.

**Conclusions:**

Most of non-antibacterials inhibited *H. pylori* isolates, similar to antibiotics but their MICs exceeded those of antibiotics and their plasma concentrations. At low plasma concentration, non-antimicrobials may act as weak antibacterials, antibiotic adjuvants and immunostimulators.

## 1 Introduction

*Helicobacter pylori* is a spiral epsilon-proteobacterium that has been evolved to colonize the gastric epithelium of humans as its unique niche. Microscopic observation of spiral bacteria in gastric epithelium was first reported in1893 in dogs [1] and thirteen years later in humans [2]. However, failure in culturing *H. pylori* from human stomach hampered identification of the bacterium and discovery of its correlation with gastric diseases. The initial cultivation of *H. pylori* from gastric biopsies of dyspeptic patients and relief after bacterial eradication with antibiotics confirmed the implication of *H. pylori* infection in gastritis [3] with the possible severe consequences as peptic ulcer and gastric cancer [4]. On the other hand, reduction in the frequency of gastric cancer in several countries with widespread use of antibiotics for treatment of different infectious diseases led to the conclusion that cure of a primary infection may also eliminate *H. pylori* from the stomach of patients [5]. Accordingly, antibiotic therapy for patients complaining of dyspepsia started with two antibiotics and a proton pump inhibitor. However, despite a high rate of success in initial antibiotic therapies, *H. pylori* started to show resistance [6] that is now a worldwide problem [7,8].

Globally reported resistance of different bacteria to a number of commonly used antibiotics has urged many investigators around the world to evaluate the antimicrobial potential of many currently used non-antimicrobial drugs. These drugs may have direct antimicrobial activity or when used in combination with antibiotics, they disrupt bacterial resistance or enhance patient's immune response [9,10]. Reports published in recent years indicate that an increasing number of investigations have focused on antibacterial properties of non-antimicrobial drugs, ranging from vitamins to antineoplastics. These drugs in addition to their typical action on targets in human body have shown promising antimicrobial activity against Gram-positive and Gram-negative bacteria [11]. It has been suggested that bacterial cell components have a common evolutionary origin with those of mammalian cells. These components that are often involved in vital activities of bacteria are the important targets of non-antibiotics. The antibacterial activity of these drugs may be non-specific, targeting multiple functions in bacterial cells or specific, inhibiting a single bacterial activity. Examples of these functions include; phosphorylation and dephosphorylation as a common signaling mechanism, initiation and propagation of electrical signaling through voltage-gated channels and metalloenzymes that have a metal center for catalytic activity [12]. Furthermore, results of several reports indicated that non-antibacterial drugs often exert their inhibitory action through disrupting of bacterial cell plasma and outer membranes [11,13,14].

There is limited number of studies on *anti*-*H. pylori* activity of non-antibacterial drugs. These studies often used a single or one group of drugs such as bismuth subsalicylate (BSS) [15,16] aspirin (ASA) [17], vitamin C (Vit C) [[18], [19], [20]], proton pump inhibitors (PPIs) [[21], [22], [23]] and antifungals [24,25]. Results of these studies showed the high potential of such drugs for successful eradication of *H. pylori*. However, there is no comprehensive study that describes the *anti*-*H. pylori* activity of a wider range of non-antibacterial drugs. On the other hand, investigators are trying to find more effective strategies to overcome *H. pylori* resistance to therapeutic regimens. In a recent study, antibiotic-free nanoparticles (NPs) were constructed by assembling metformin-linoleic acid (ML) and linoleic acid (LA) NPs that encapsulated urease inhibitor ebselen (EB) and was coated by fucoidan (FU). The FU/ML-LA/EB NPs destroyed *H. pylori* biofilm in cultured human gastric mucosal epithelial cells, killed dispersed bacteria by inhibiting urease and when taken up by epithelial cells, caused lysosomal acidification and activation of adenosine monophosphate-activated protein kinase (AMPK) that degraded intracellular *H. pylori*. Furthermore, EB and LA fractions in NPs with antioxidant properties alleviated the excessive oxidative stress in response to *H. pylori* infection and its severe consequences such as gastric mucosal damage [26].

In this study, antibacterial efficacy of nineteen commonly used non-antimicrobial drugs and vitamins against 20 *H. pylori* was investigated in order to evaluate whether consumption of such compounds for treatment of non-infectious diseases exert effective antimicrobial activity against *H. pylori* in the stomach and reduce the risk of development of severe gastric diseases. These drugs included 3 proton pump inhibitors; omeprazole (OMP), lansoprazole (LPZ) and pantoprazole (PAN), 3H_2_-blockers_;_ famotidine (FAM), cimetidine (CIM) and ranitidine (RAN), bismuth subsalicylate (BSS), 3 antifungals; ketoconazole (KTC), fluconazole (FLC) and amphotericin B (AMB), 3 statins; atorvastatin (ATV), simvastatin (SMV) and rosuvastatin (RSV), acetaminophen (ACE), aspirin (Acetylsalicylic acid, ASA), vitamin B1(Vit B1, thiamine), vitamin B6 (Vit B6, pyridoxine) and vitamin B_complex_ (Vit B_complex_) and vitamin C (Vit C, l-ascorbic acid). The 4 commonly used *anti*-*H. pylori* antibiotics; metronidazole (MTZ), clarithromycin (CLR), amoxicillin (AMX) and tetracycline (TET) were also examined for comparison. The range of concentrations used for drugs included their published minimum inhibitory concentration (MIC). Furthermore, plasma concentration of drugs in the body of normal individuals was used for comparison. Information related to mechanism of action of drugs and the susceptible bacteria are summarized in Table 1.

**Table 1:** Summarized properties of 19 non-antibacterial and 4 antibiotics; diseases used for, mechanism of action in humans, mechanism of anti-microbial action and susceptible bacteria.

Drug name	Diseases used for	Mechanism of action in humans	Mechanism of anti-microbial action	Susceptible bacteria
MTZ	Oral infections and parasite related diseases [27].	–	Breakage in DNA strands and inhibition of nucleic acid synthesis [27].	Anaerobic and facultative anaerobic bacteria [27].
CLR	Respiratory infections and skin and soft tissue infections [28].	–	Inhibition of protein synthesis by interfering with aminoacyl translocation in ribosomes [28].	Gram-positive and Gram-negative bacteria [28].
AMX	Respiratory and urinary tract infections [29].	–	Inhibition of peptidoglycan synthesis [29].	Gram-positive and Gram-negative bacteria [29].
TET	Respiratory and bowel diseases and syphilis infections [30].	–	Inhibition of protein synthesis by preventing the attachment of charged aminoacyl-tRNA to the A-site on the ribosomes [30].	Gram-positive and Gram-negative bacteria [30].
PPIs	Acid reflux disease, gastric bleeding and *Helicobacter pylori* infection [31].	Inhibition of proton pumps (H^+^/K^+^ ATPases) of gastric epithelial cells [31].	Inhibition of bacterial growth and replication with unknown mechanism [31].	*H. pylori* [21], *Lactobacillus gasseri* and *Streptococcus gordonii* [32].
H_2-_ blockers	Acid-peptic diseases and *H. pylori* infection [33].	Reducing gastric acid secretion by blocking H_2_ - receptors in parietal cells and inhibiting human carbonic anhydrase [33].	Inhibition of carbonic anhydrase and bacterial growth [33].	*Escherichia coli* and *Staphylococcus aureus* [34].
BSS	Gastrointestinal infectious diseases and *H. pylori* infection [35].	Increasing mucosal protective factors, including prostaglandin with no toxicity due to low absorption in mammalian cells [35].	Forming complexes with bacterial outer membrane, causing cell lysis. Inhibiting bacterial urease, catalase and alcohol dehydrogenase [35].	*H. pylori* [15]*, Clostridium difficile*, *E. coli* and *S. aureus* [36].
KTC/FLC	Fungal infections [37].	Ketoconazole inhibits the enzymes involved in steroid synthesis, causing adrenal insufficiency. It can also cause severe liver injury [37].	Depletion of ergosterol from fungal plasma cell membrane that alters its structure and function. Bacterial lysis due to inhibition of cholesterol incorporation into cell membrane [25].	*H. pylori* [25] and *S. aureus* [38].
AMB	Fungal infections [39].	Interacts with cholesterol in human cell membrane, leading to loss of minerals, anaphylaxia, fever and renal failure [39].	Interacts with sterols in fungal cell membrane causing cytoplasmic leakage and death [39].	–
Statins	Hypercholesterolemia and inflammatory diseases [40].	Inhibit HMG-CoA reductase^†^. Anti-inflammatory, immunomodulatory and antioxidant [40,41].	Reduction of cholesterol availability to *H. pylori* with negative effect on bacterial colonization, innate immune evasion and antibiotic resistance [42].	*Haemophilus influenzae* and methicillin-resistant *S. aureus* [43].
ACE	Antipyretic and Analgesic [44].	Inhibits cyclooxygenase −1 variant enzyme in central nervous system [44].	Inhibition of bacterial growth and replication with unknown mechanism [45].	*S*. *aureus* and *Paracoccus yeei* [45].
ASA	Antipyretic, Analgesic and anti-inflammatory [46].	By inhibition cyclooxygenase activity, causes inhibition of prostaglandin formation and thus reduction of inflammation, swelling, pain and fever [46].	Inhibition of bacterial growth and replication with unknown mechanism.	*H. pylori* [17] and *Klebsiella pneumoniae* [47].
B-Vit	Food supplement for improving human health [48].	Involved in vital activities of all living cells [48], with immunomodulatory property that helps to fight the pathogens [49].	Antibacterial effect with unknown mechanism [50].	*Salmonella* spp*., Listeria* spp*.* and *E. coli* [51].
Vit C	Food supplement for improving human health [52].	Serves as an antioxidant and a cofactor of several enzymes. Also enhances immune response and iron absorption [52].	Inhibition of bacterial growth and replication with unknown mechanism.	*H. pylori* [19] and *Mycobacterium tuberculosis* [53].

MTZ, Metronidazole; CLR, Clarithromycin; AMX, Amoxicillin; TET, Tetracycline; PPIs, Proton Pump Inhibitors; BSS, Bismuth salt; KTC, Ketoconazole; FLC, Fluconazole; AMB, Amphotericin B; ACE, Acetaminophen; ASA, Acetylsalicylic acid; B-Vit, B-vitamins; Vit C, Vitamin C; †HMG-CoA reductase, 3-hydroxy-3-methylglutaryl-coenzyme A reductase.

## 2 Materials and methods

### 2.1 Patients and *H. pylori* isolates

Twenty *H. pylori* strains used in this study were isolated from gastric biopsies of 20 dyspeptic patients that exhibited positive rapid urease test. Patients included 9 women (45%) and 11 men (55%) with the age range of 7–86 yr. They were referrals to the endoscopy unit of digestive disease research institute, Shariati hospital, Tehran, Iran. All patients signed informed consent and the study was approved by the research ethics committee of Tehran University of Medical Sciences (code number: IR. TUMS.DDRI.REC.1400.023). To obtain reliable results from *H. pylori* culture, patients were recommended to stop taking proton pump inhibitor and antibiotics 2 weeks before endoscopy [54]. Gastric biopsies were cultured on selective brucella agar (Pronadisa, Spain), supplemented with 7% defibrinated sheep blood, vancomycin (10 mg/L), trimethoprim (5 mg/L), polymyxin B (50 μg/L) and amphotericin B (4 mg/L). All the used antibiotics were purchased from Sigma Company (Sigma, Germany). Cultures were examined for *H. pylori* growth after 3–5 days incubation under microaerobic atmosphere at 37 °C. Out of 20 bacterial isolates, 19 produced glistening pin-pointed colonies on blood agar and examination of their Gram-stained smears with the light microscope showed Gram-negative spirals. The # 20 bacterial isolate showed mucoid growth on blood agar and appeared as Gram-negative bacteria with spiral morphology when examined with the light microscope. All the 20 bacterial isolates exhibited positive catalase, oxidase, and urease activities.

### 2.2 Molecular identification of *H. pylori* isolates

The identity of all isolated bacteria as *H. pylori* was confirmed by detection of *H. pylori*-specific 16S rDNA. Bacterial DNAs were extracted using phenol-chloroform method and amplification of *H. pylori*-specific 16S rDNA was performed with HP1 (5′-GCAATCAGCGTCAGTAATGTTC-3′) and HP2 (5′-GCTAAGAGA TCAGCCTATGTCC-3′) primers [55], as previously described [56]. PCR products were electrophoresed on 1% agarose gel and amplicons of isolates # 19 and 20 were sequenced and matched with reference sequences in the GenBank by BLAST program.

### 2.3 Preparation of blood agar plates containing 19 non-antibacterial drugs and 4 antibiotics metronidazole, clarithromycin, amoxicillin and tetracycline

Agar dilution method was used for susceptibility testing of *H. pylori* according to CLSI approved methodology [57]. A range of concentrations (μg/mL) was used for each drug, including their published MIC. The four used antibiotics (Sigma, Germany) and their MICs included MTZ (8 μg/mL), CLR (2 μg/mL), AMX (1 μg/mL) and TET (0.5 μg/mL). These MICs were determined in our previous studies after performing susceptibility tests with a range of concentrations for MTZ (4, 8, 16 and 32 μg/mL), CLR (0.25, 0.5, 1 and 2 μg/mL), AMX (0.25, 0.5 and 1 μg/mL), and TET (0.25, 0.5 and 1 μg/mL) [58]. Selected MICs for antibiotics were almost similarly reported in other studies around the globe [59,60]. Tested non-antibacterials included PPIs (Arya, Iran); OMP, LPZ and PAN (8, 16, 32, 64 and 128 μg/mL) [21], H_2_-blockers (Arya, Iran); FAM, CIM and RAN (1000, 2000, 4000 and 8000 μg/mL) [34], BSS (Arya, Iran) (5, 10, 15 and 20 μg/mL) [35], antifungals (Arasto, Iran); KTC, FLC and AMB (32, 64, 128 and 256 μg/mL) [24,25], statins (Darou Pakhsh, Iran); ATV, SMV and RSV (50, 100, 150, 200, 250 and 500 μg/mL) [43], ACE (Darou Pakhsh, Iran) (1.25, 2.5, 5, 10, 20 and 40 μg/mL), ASA (Darou Pakhsh, Iran) (100, 200, 400 and 800 μg/mL) [17], B-vitamins (Darou Pakhsh, Iran); Vit B1, Vit B6 and Vit B_complex_ (2500, 5000, 10,000 and 20,000 μg/mL) [51] and Vit C (Darou Pakhsh, Iran) (512, 1024 and 2048 μg/mL) [18]. Fresh (100%) dimethyl sulfoxide (DMSO, Merck-Germany) was used for preparation of drugs' solutions [21,43,58]. Blood agar plates containing different concentrations of drugs were prepared by adding appropriate volumes of concentrated stock solutions to blood agar. All drugs’ solutions were filter-sterilized, using 0.22 μm nitrocellulose membrane filters (Control Biogen, Spain). Autoclaved brucella agar base was cooled to ∼45 °C and 7–10% sheep blood was added and swirled to become homogenized. Finally, filter-sterilized drugs were added to cool (<45 °C) blood agar, mixed by swirling and dispensed into sterile plates. It is noteworthy that all the drugs exhibited antibacterial activity after being added to cool blood agar. All *H. pylori* isolates exhibited confluent growth when cultured on blood agar as well as blood agar containing DMSO with the final concentration of 2.5%, showing lack of DMSO toxicity to *H. pylori*. Our search for finding MIC for acetaminophen was not successful. Accordingly, we used concentrations of 1.25–40 μg/mL that included the MICs of the used antibiotics.

### 2.4 *S*usceptibility tests for 20 *H. pylori* isolates

Fresh sub-cultures of all 20 *H. pylori* isolates on blood agar were used for preparation of bacterial suspensions with the turbidity of MacFarland standard No.2 (6 × 10^8^ cell/mL). A 10-μL volume of each bacterial suspension was spot-inoculated on the blood agar containing different concentrations of drugs. Plates were incubated as mentioned earlier and examined after 3–5 days. All the susceptibility tests were performed in triplicates and the results were recorded as susceptible (no growth) or resistant (growth).

### 2.5 Synergistic antibacterial effect of MTZ and SMV on mucoid *H. pylori*

The mucoid *H. pylori* (#20) that showed resistance to 19 out of 23 drugs, including SMV and MTZ was examined for the synergistic antibacterial effect of MTZ and SMV, using combined disc diffusion and agar dilution methods [61]. First, *H. pylori* susceptibility to MTZ and SMV was tested by disc diffusion and agar dilution method, respectively. Bacterial suspension with the turbidity of MacFarland standard No.2 was surface-inoculated on blood agar plates and sterile blank paper discs were superimposed on their surface and impregnated with different concentrations of MTZ (8, 4, 2 and 1 μg/mL). Second, different concentrations of SMV were added to cooled blood agar to reach the final concentrations of 100, 50, 25 and 12.5 μg/mL. SMV-containing plates were inoculated with 6 *H. pylori* isolates, including 5 non-mucoids (No:1–5) and the mucoid one (No:6). Third, combined susceptibility test was performed by first surface-inoculating the bacterial suspension on the blood agar plates containing 100, 50, 25 and 12.5 μg/mL of SMV and then superimposing the blank paper discs impregnated with different concentrations of MTZ (8 and 4 μg/mL). Drugs’ combinations included: (8 μg/mL MTZ +100 μg/mL SMV), (8 μg/mL MTZ +50 μg/mL SMV), (4 μg/mL MTZ +25 μg/mL SMV) and (4 μg/mL MTZ +12.5 μg/mL SMV). Blood agar with no added drug was used as a control. Plates were incubated as mentioned earlier and examined after 3–5 days.

### 2.6 Statistical analysis

Non-parametric tests, including the Chi-Square and Mann-Whitney U were employed to compare various groups within our research. For our statistical analysis, we relied on IBM SPSS Statistics version 26. *P*-value <0.05 was considered as significant.

## 3 Results

### 3.1 Molecular identification of *H. pylori* isolates

**Fig. 1:**
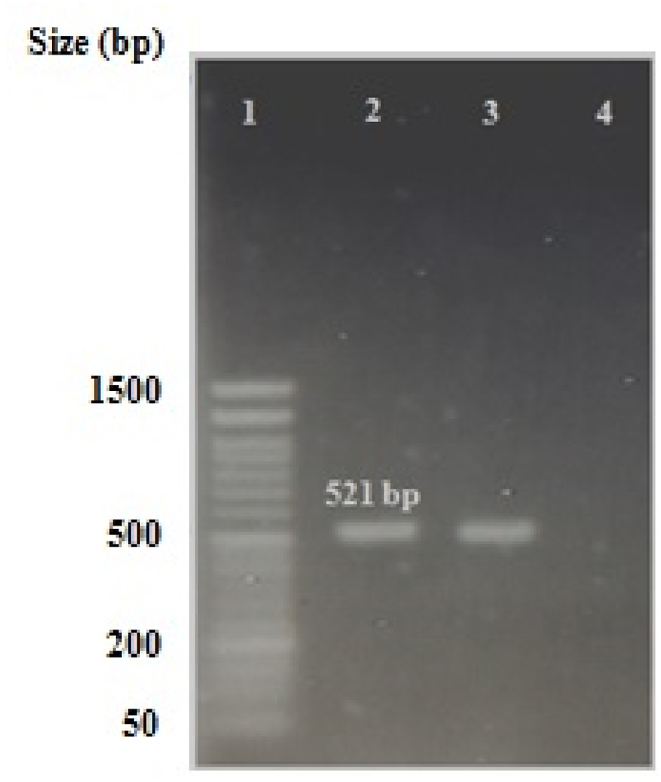
Detection of 16S rDNA in *H. pylori* isolates # 19 and # 20. Lane 1: Molecular ladder (50 bp), lane 2: 521 bp product from spiral *H. pylori*, lane 3: 521 bp product from mucoid *H. pylori* and lane 4: No template. Full and non-adjusted image of Fig. 1 is provided as supplementary material.

Electrophoresis of PCR products amplified from all *H. pylori* isolates showed amplicons with the size of 521 bp. Amplicons of isolates #19 and #20 (Fig. 1) were sequenced and matched with sequences in GenBank by the BLAST program. Results of BLAST analysis showed 99%–100% sequence homology between amplified fragments from isolates #19 and #20 and the corresponding sequences of the reference *H pylori* in GenBank, confirming the identity of isolates as *H. pylori.*

### 3.2 Susceptibility of 20 *H. pylori* isolates to 23 drugs

A considerable number *H. pylori* isolates showed susceptibility to currently used antibiotics: MTZ [8 μg/mL] (16, 80%), CLR [2 μg/mL] (18, 90%), AMX [1 μg/mL] (20, 100%) and TET [0.5 μg/mL] (14, 70%). *H. pylori* isolates also showed a considerable susceptibility to non-antibacterial drugs; PPIs: OMP [32 μg/mL], LPZ [8 μg/mL] and PAN [128 μg/mL] (16, 80%), H_2_-blockers: FMT [2000 μg/mL] (15, 75%), CIM [2000 μg/mL] (16, 80%) and RAN [8000 μg/mL] (16, 80%), BSS [15 μg/mL] (17, 85%), antifungals: KTC [64 μg/mL] (16, 80%), FLC [256 μg/mL] (6, 30%) and AMB [128 μg/mL] (0, 0%), statins: ATV [250 μg/mL] (17, 85%), SMV [100 μg/mL] (18, 90%) and RSV [200 μg/mL] (7, 35%), ACE [40 μg/mL] (15, 75%), ASA [800 μg/mL] (15, 75%), B-vitamins: Vit B1 [10,000 μg/mL] (20, 100%), Vit B6 [5000 μg/mL] (20, 100%) and Vit B_complex_ [20,000 μg/mL] (16, 80%) and Vit C [2048 μg/mL] (17, 85%) (Table 2).

**Table 2:** Susceptibility of 20 *H. pylori* isolates to 19 non-antibacterial and 4 antibiotics, their minimum inhibitory concentration (MIC) and plasma concentration.

Drug name	No. of susceptible isolates (%)	MIC in this study (μg/mL)	MIC in other studies (μg/mL)	Plasma concentration (μg/mL)
MTZ	16 (80)	8	4-12 [[58], [59], [60]]	–
CLR	18 (90)	2	0.25–2 [[58], [59], [60]]	–
AMX	20 (100)	1	0.25–1 [[58], [59], [60]]	–
TET	14 (70)	0.5	0.25–1 [[58], [59], [60]]	–
OMP	16 (80)	32	6.25–32 [21]	8 [31]
LPZ	16 (80)	8	8 [21]	1 [31]
PAN	16 (80)	128	20-1000 [32]	3.3 [31]
FMT	15 (75)	2000	8000-64000 [34]	0.02 [62]
CIM	16 (80)	2000	–	1.5 [63]
RAN	16 (80)	8000	–	0.898 [64]
BSS	17 (85)	15	4-32 [36]	0.05 [16]
KTC	16 (80)	64	8–32 [24,25]	3–4.5 [65]
FLC	6 (30)	256	–	0.5–9 [66]
AMB	0 (0)	128	–	0.136–0.5 [67]
ATV	17 (85)	250	41-250 [43]	0.01–0.3 [41]
SMV	18 (90)	100	146-292 [43]	0.01–0.3 [41]
RSV	7 (35)	200	100-500 [43]	0.01–0.3 [41]
ACE	15 (75)	40	1250 [45]	10-20 [68]
ASA	15 (75)	800	100-512 [17]	20-200 [69]
Vit B1	20 (100)	10,000	20,000 [51]	0.004 [70]
Vit B6	20 (100)	5000	–	>0.0074 [71]
Vit B_complex_	16 (80)	20,000	–	–
Vit C	17 (85)	2048	150-20000 [19]	>4 [72]

MTZ, Metronidazole; CLR, Clarithromycin; AMX, Amoxicillin; TET, Tetracycline; OMP, Omeprazole; LPZ, Lansoprazole; PAN, Pantoprazole; FMT, Famotidine; CIM, Cimetidine; RAN, Ranitidine; BSS, Bismuth salt; KTC, Ketoconazole; FLC, Fluconazole; AMB, Amphotericin B; ATV, Atorvastatin; SMV, Simvastatin; RSV, Rosuvastatin; ACE, Acetaminophen; ASA, Acetylsalicylic acid; Vit B_1_, Vitamin B_1_; Vit B_6_, Vitamin B_6_; Vit B_complex_, Vitamin B_complex_; Vit C, Vitamin C.

### 3.3 Classification of *H. pylori* isolates according to their susceptibility or resistance to 23 drugs, 4 antibiotics and 19 non-antibacterials

The 20 *H. pylor*i isolates were classified into 9 groups according to the number of anti-bacterial drugs they showed susceptibility to: Three isolates (15%) were susceptible to 21 drugs, 4 (20%) to 20, 2 (10%) to 19, 4 (20%) to 18, 3 (15%) to 17, 1 (5%) to 16, 1 (5%) to 14, 1 (5%) to 9 and 1 (5%) to 4. The highest susceptibility rate (100%) belonged to AMX, Vit B1and Vit B6 followed by CLR (90%) and SMV (90%) (Table 3). In the other words, 3 isolates (15%) showed resistance to 2 drugs, 4 (20%) to 3, 2 (10%) to 4, 4 (20%) to 5, 3 (15%) to 6, 1 (5%) to 7, 1 (5%) to 9, 1 (5%) to 14 and 1 (5%) to 19 (Table 3). The highest resistance rate (100%) belonged to AMB, followed by FLC (70%), RSV (65%) and TET (30%). Overall, statistical analysis showed that susceptibility of 20 *H. pylori* isolates to 16 out of 19 non-antimicrobials (75–100%) was not significantly different from their susceptibility to 4 antibiotics (70–100%) (*P*-value 0.45). The highest susceptibility rate (100%) belonged to Vit B1, Vit B6 and AMX. Furthermore, out of 20 *H. pylori* isolates, 17 (85%) were susceptible to ≥13 non-antimicrobials and 3 (15%) were susceptible to < 13 (*P*-value 0.003).

**Table 3:** Classification of 20 *H. pylori* isolates into 9 groups according to the number of drugs they showed susceptibility (+) or resistance (−) to.

MDS-grs	# of isolates	AMX	V B1	VB6	CLR	SMV	ATV	VC	BSS	MTZ	OMP	LPZ	PAN	CIM	RAN	KTZ	V B_C_	FAM	ACE	ASA	TET	RSV	FLC	AMB
21	3	+	+	+	+	+	+	+	+	+	+	+	+	+	+	+	+	+	+	+	–	+	+	–
		+	+	+	+	+	+	+	+	+	+	+	+	+	+	+	+	+	+	+	+	–	+	–
		+	+	+	+	+	+	+	+	+	+	+	+	+	+	+	+	+	+	–	+	+	+	–
20	4	+	+	+	+	+	+	+	+	+	–	+	+	+	+	+	+	+	+	+	+	+	–	–
		+	+	+	+	+	+	+	+	+	+	+	**-**	+	+	+	+	+	+	+	+	+	**-**	**-**
		+	+	+	+	+	+	+	+	+	+	–	+	+	+	+	+	+	+	+	+	–	+	–
		+	+	+	+	+	+	+	+	+	+	+	+	+	–	+	+	+	+	+	+	–	+	–
19	2	+	+	+	+	+	+	+	+	+	+	–	+	+	+	+	+	+	+	+	+	–	–	–
		+	+	+	+	+	+	+	+	+	+	+	+	+	+	+	+	+	+	+	–	–	–	–
18	4	+	+	+	+	+	+	+	+	+	+	+	–	+	+	+	–	+	+	+	+	–	–	–
		+	+	+	+	+	+	+	+	+	+	+	+	+	+	+	+	+	–	–	+	–	–	–
		+	+	+	–	+	+	+	+	+	+	+	+	+	–	+	+	+	+	–	+	+	–	–
		+	+	+	+	+	+	+	–	+	+	+	+	+	+	+	+	–	–	+	+	+	–	–
17	3	+	+	+	+	+	+	+	+	–	+	+	+	+	+	–	+	+	–	+	+	–	–	–
		+	+	+	+	+	+	+	+	+	+	+	+	–	+	+	–	–	+	+	+	–	–	–
		+	+	+	–	+	+	–	+	+	–	+	+	+	+	+	–	+	+	+	+	–	+	–
16	1	+	+	+	+	+	+	+	–	+	+	–	+	–	+	+	+	–	+	+	–	+	–	–
14	1	+	+	+	+	+	–	+	+	–	–	+	+	+	+	–	+	+	–	+	–	–	–	–
9	1	+	+	+	+	–	–	–	+	–	+	+	–	–	–	–	+	–	+	–	–	–	–	–
4	1	+	+	+	+	–	–	–	–	–	–	–	–	–	–	–	–	–	–	–	–	–	–	–

MDS-grs, Multidrug susceptible groups; AMX, Amoxicillin; VB_1_, Vitamin B_1_; VB_6_, Vitamin B_6_; CLR, Clarithromycin; SMV, Simvastatin; ATV, Atorvastatin; VC, Vitamin C; BSS, Bismuth salt; MTZ, Metronidazole; OMP, Omeprazole; LPZ, Lansoprazole; PAN, Pantoprazole; CIM, Cimetidine; RAN, Ranitidine; KTC, Ketoconazole; VB_c_, Vitamin B_complex_; FMT, Famotidine; ACE, Acetaminophen; ASA, Acetylsalicylic acid; TET, Tetracycline; RSV, Rosuvastatin; FLC, Fluconazole; AMB, Amphotericin B; +, Susceptible; -, Resistant.

### 3.4 Synergistic antibacterial effect of MTZ and SMV on mucoid *H. pylori*

**Fig. 2:**
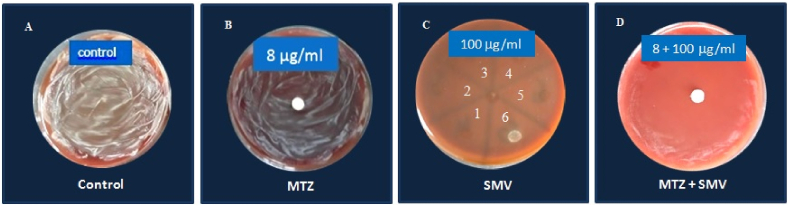
Susceptibility of mucoid *H. pylori* to metronidazole (MTZ 8 μg/mL) and simvastatin (SMV 100 μg/mL). From left to right: A) Control mucoid *H. pylori* with confluent growth. B) Mucoid *H. pylori* showed resistance when treated with MTZ alone, by disc diffusion method. C) Mucoid *H. pylori* (6) showed resistance when treated with SMV alone, by agar dilution. Non-mucoid *H. pylori* isolates (1–5) were susceptible to SMV. D) Mucoid *H. pylori* was inhibited by combination of MTZ and SMV.

Similar to the control mucoid *H. pylori* with confluent growth (Fig. 2A), mucoid *H. pylori* showed resistance and confluent growth when treated with MTZ (1–8 μg/mL) alone (Fig. 2B). Compared with non-mucoid *H. pylori* isolates (No:1–5) that were inhibited with SMV (12.5–100 μg/mL), mucoid *H. pylori* (No:6) showed resistance (Fig. 2C). However, mucoid *H. pylori* showed a considerable susceptibility to MTZ (8 μg/mL) combined with SMV (100 μg/Ml) with the inhibition zone diameter of 60 mm (Fig. 2D). No synergistic inhibition was observed with other combinations of MTZ and SMV.

## 4 Discussion

Several studies have focused on the problem of antimicrobial resistance of bacterial pathogens, examining the antimicrobial and immunological properties of different non-antibacterial drugs alone or in combination with antibiotics. Although a considerable number of investigators have succeeded to show the antibacterial effect of non-antibacterial drugs on a wide range of pathogenic bacteria, the mechanism of action of these drugs has remained largely unknown. It has been suggested that non-antibiotics interact with common prokaryotic and eukaryotic targets because of their common evolutionary origin [12]. However, there is no report to show that results of *in vitro* studies correspond with those of *in vivo* studies. In this study the antibacterial effect of 19 non-antibiotic drugs and 4 currently used antibiotics against *H. pylori* isolates was examined to propose that when these drugs are used for treatment of non-infectious diseases, they may exert effective antibacterial activity on the existing bacterial infections such as that of *H. pylori*.

The three PPIs examined, inhibited the growth of 80% of *H. pylori* isolates, although with MICs of increasing order; LPZ (8 μg/mL), OMP (32 μg/mL) and PAN (128 μg/mL). In a study by our group, OMP (32 μg/mL) and LPZ (8 μg/mL) but not PAN (128 μg/mL) inhibited the growth of 78.6% of *H. pylori* isolates [21]. Results of a study showed that when patients used PPI along with antibiotics for eradication of *H. pylori*, spiral bacteria turned into coccoid morphology [22] and showed reduced urease activity and virulence [23]. In one study, OMP with MIC of 200 μg/mL exhibited antibacterial activity against *Staphylococcus aureus* and *Escherichia coli* [73]. The MICs of PPIs obtained in the present study (8–128 μg/mL) were higher than their related plasma concentration; OMP (8 μg/mL), LPZ (1 μg/mL) and PAN (3.3 μg/mL) [31].

FMT and CIM at MIC 2000 μg/mL inhibited 75% and 80% of *H. pylori* isolates, respectively. However, RAN inhibited 80% of the isolates with 4-times higher concentration (8000 μg/mL). FMT with the MIC of 8000–64000 μg/mL exhibited a significant antibacterial activity against *E. coli*, *S. aureus* and *Pseudomonas aeruginosa* while RAN and CIM showed no inhibitory effect [34]. MICs of H_2_- blockers obtained in this study (2000–8000 μg/mL) were much higher than their related plasma concentration (μg/mL): FMT 0.02 [62], CIM 1.5 [63] and RAN 0.896 [64]. Results of a recent study have indicated that antibacterial effect of FAM combined with rifampicin on *Acinetobacter baumannii* and *Salmonella typhimurium* was more effective than when they were used alone [74]. It was suggested that FMT by disrupting bacterial cell outer membrane in *A. baumannii*, exerted a negative impact on bacterial virulence, drug resistance (efflux pump) [75], and release of damaging inflammatory factors [76]. Furthermore, inhibition of efflux pump in *A. baumannii* by usnic acid, turned the tigecycline-resistant bacteria to susceptible [77].

BSS with the concentration of 15 μg/mL inhibited 85% of *H. pylori* isolates. This inhibitory concentration of BSS was among the lowest effective antibacterial concentrations found in this study, after antibiotics (0.5–8 μg/mL) and LPZ (8 μg/mL). Results of a study demonstrated the inhibitory effect of BSS on *H. pylori* growth with the concentration of 4–8 μg/mL [15]. Since *H. pylori* does not exhibit primary and secondary resistance to BSS, it is often included in different therapeutic regimens for effective eradication of antibiotic-resistant *H. pylori* [16]. The MIC obtained for BSS in this study (15 μg/mL) was much higher than its related plasma concentration (0.05 μg/mL) [16]. Synergistically-enhanced antibacterial activity of BSS-norfloxacin [78] and BSS-ciprofloxacin has been reported against different bacteria, including *E. coli* and *S. aureus.* [36].

All the 20 *H. pylori* isolates were resistant to AMB however, 80% were susceptible to KTC (64 μg/mL) and 30% to FLC (256 μg/mL). Results of a similar study showed that KTC inhibited *H. pylori* with the MIC range of 8–32 μg/mL [24]. In a study by our group, KTC inhibited the growth of MTZ-resistant *H. pylori* with the MIC of 8 μg/mL [25]. The MICs found for KTC (64 μg/mL) and FLC (256 μg/mL) in this study exceeded their respective plasma concentrations, KTC 3–4.5 μg/mL [65] and FLC 0.5–9 μg/mL [66]. Reports showed that azole antifungal could enhance the antibacterial effectiveness of TET against methicillin-resistant *S. aureus* [79]. Furthermore, KTC could enhance the antibacterial activity of fluoroquinolones against multi-drug resistant *S. aureus* by inhibiting efflux pump and biofilm formation [38].

SMV (100 μg/mL) inhibited 90% of *H. pylori* isolates, ATV (250 μg/mL) 85% and RVS (200 μg/mL) 35%. Reports indicated the inhibitory action of SMV on *Haemophilus influenzae* with the MIC of 146 to >250 μg/mL and on *Streptococcus pneumoniae* with the MIC of 292 μg/mL [43]. Furthermore, statins inhibited the methicillin-resistant *S. aureus* and vancomycin-resistant Enterococci [80]. The MICs of statins determined in the present study (100–250 μg/mL) far exceeded their peak plasma concentration, ranged between 0.01 and 0.3 μg/mL [41]. Association of SMV with sub-inhibitory concentration of colistin resulted in their synergistic effect against *A. baumannii*, *E. coli*, *Klebsiella pneumoniae* and *P. aeruginosa* while the MIC of SMV was reduced from >265 μg/mL to a range of 8–32 μg/mL [81].

Among the 20 *H. pylori* isolates studied, 75% showed susceptibility to ACE at 40 μg/mL. ACE inhibited the growth of *S. aureus* and *Paracoccus yeei* at 1250 μg/mL while *Enterobacter aerogenes* and *Enterobacter cloacae* exhibited resistance [45]. The MICs of ACE determined against *H. pylori* in this study (40 μg/mL) and against other bacteria in Al-Janabi report (1250 μg/mL) [45] exceeded the reported plasma concentration of ACE (10–20 μg/mL) [68]. Similarly, 75% of *H. pylori* isolates showed susceptibility to ASA at 800 μg/mL. ASA at concentrations (70–4000 μg/mL) higher than ACE (1250 μg/mL) showed antibacterial activity on different bacteria, including *A. baumannii* and *E. coli* [14]. ASA at its therapeutic plasma level (100 μg/mL) inhibited the growth of *H. pylori* [17] and at 50–200 μg/mL inhibited other bacteria, including *K. pneumoniae* [47]. The MIC of ASA for 66 *H. pylori* strains was determined as a range of 256–512 μg/mL [17]. Furthermore, ASA showed negative impact on *H. pylori* urease and vacuolating cytotoxin A activities [82]. The MIC of ASA found against *H. pylori* in this study (800 μg/mL) was higher than its reported plasma concentration (20–200 μg/mL) [69]. It has been demonstrated that ASA while inhibiting the growth of *H. pylori* at MIC 512 μg/mL, increased its susceptibility to AMX, CLR and MTZ at 180 μg/mL [17].

B-vitamins showed antibacterial activity against *H. pylori* at considerably high concentrations. Vit B1 (10,000 μg/mL) and Vit B6 (5000 μg/mL) inhibited 100% of *H. pylori* isolates while Vit B_complex_ (20,000 μg/mL) inhibited 80% of isolates. In a study, riboflavin (Vit B2) at concentration of 50,000 μg/mL inhibited the growth of *S. aureus* and *P. aeruginosa* [50]. The MIC of B-vitamins found against *H. pylori* in this study (5000–20000 μg/mL) far exceeded their reported plasma concentrations (0.004 μg/mL - >0.0074 μg/mL) [70,71]. In a study, Vit B1 and Vit B6 exhibited synergistic antibacterial activity when combined with tazobactam, polymixin B and doripenem. Similarly, Vit B12 exhibited synergistic antibacterial activity when combined with tazobactam and polymixin B and Vit B2 when combined with polymixin B [61]. Reports have described the important role of Vit B1 and Vit B6 as immune system stimulators. Vit B1 exhibits anti-inflammatory effect and suppresses oxidative stress-induced NF-κB activation, while Vit B6, plays an important role in activating IFN-γ that mediates cellular immune activation [83].

Vit C inhibited 85% of *H. pylori* isolates at concentration of 2048 μg/mL. This antibacterial effect of Vit C could be due to the activity of Vit C oxidation products, l-dehydroascorbic acid or l-diketogluconic acid as described in an *in vitro* study on *Campylobacter jejuni* [84]. In a study on 64 *H. pylori* isolates, 90% were inhibited at Vit C concentration of 2048 μg/mL [18]. In another study, Vit C at 10,000–20,000 μg/mL effectively inhibited *H. pylori* growth [19]. Similarly, antibacterial activity of Vit C was determined as 5000–20000 μg/mL against *K. pneumoniae* and *E. coli* [85]. Vilchèze et al. reported that l-ascorbic acid killed the strains of *Mycobacterium tuberculosis* that were resistant to a wide range of antibiotics [53]. Furthermore, the synergistic effect of l-ascorbic acid combined with deferoxamine has been found against *Proteus mirabilis* and *S. aureus* [86]. The *anti*-*H. pylori* MIC of Vit C determined in this study (2048 μg/mL) exceeded its reported plasma concentration (>4 μg/mL) [72]. In this regard, secretion of Vit C from plasma to gastric juice that reaches 0–28.8 μg/mL [18] and is 3–7 times higher than plasma levels [20] might play a protective role against *H. pylori.* On the other hand, in an *in vitro* study, it has been demonstrated that treatment with Vit C could interfere with development of dormant multidrug resistant *H. pylori* that emerged either as viable but nonculturable (VBNC) due to nutrient starvation or as persister when treated with 10× AMX. This interference led to bacterial resuscitation and increased susceptibility to antibiotics. Accordingly, pretreatment with Vit C was proposed for selection of vegetative forms of *H. pylori* that show higher susceptibility to antibacterial regimens [87].

The mucoid *H. pylori* that showed susceptibility to AMX, CLR, Vit B1 and Vit B6 was effectively inhibited when treated with 8 μg/mL of MTZ combined with 100 μg/mL of SMV. In our previous studies we have reported isolation of 3 mucoid *H. pylori* strains from gastric biopsies of dyspeptic patients, two with spiral [88] and one with coccoid [89] morphology, showing resistance to currently used antibiotics. It was revealed that accumulation of cholesterol and production of exopolysaccharides in mucoid *H. pylori* could lead to decrease in antibiotic uptake and emergence of resistance [90]. Further results revealed that compared with non-mucoid *H. pylori* isolates, mucoid *H. pylori* besides accumulating high amounts of cholesterol, showed high content of unsaturated fatty acids (82%). Unsaturated fatty acids in bacteria have been implicated in antioxidant activity [91] and antibiotic efflux [92]. In the present study, inhibition of mucoid *H. pylori* with a combination of high concentrations of MTZ (8 μg/mL) and SMV (100 μg/mL) might indicate the disruption of permeability barrier of bacterial cell that led to the synergistic effect of both drugs. It has been demonstrated that *H. pylori* uses cholesteryl-α- glucoside transferase (CGT) to glycosylate the exogenous cholesterol and then incorporate the glycosylated cholesterol into its cell membrane to maintain its spiral morphology and cell wall integrity. However, any alteration in cholesteryl glucoside content affects *H. pylori* morphology and renders the bacterium susceptible to various antibiotics. It has also been demonstrated that depletion of cholesteryl glucoside in *H. pylori* due to deletion of CGT-encoding gene, *hp*0421 or bacterial cultivation in the absence of cholesterol led to a significant increase in cell membrane permeability and susceptibility to antibiotics [93]. Statins are renowned for their ability to lower cholesterol by binding to the active site of HMG-CoA reductase, a rate-limiting enzyme involved in cholesterol biosynthesis [94]. Statins are also known for their anti-inflammatory, immunomodulatory, anticancer and antimicrobial effects. Although the mechanisms of their action on humans are well studied, their target (s) in bacterial cell has remained unknown [41].

## 5 Conclusion

Results of this study showed that susceptibility of 20 *H. pylori* isolates to 16 out of 19 non-antimicrobials (75–100%) was almost similar to their susceptibility to 4 antibiotics (70–100%) (*P*-value >0.05). The highest susceptibility rate (100%) belonged to Vit B1, Vit B6 and AMX followed by CLR (90%) and SMV (90%) and the highest resistance rate (100%) belonged to AMB, followed by FLC (70%), RSV (65%) and TET (30%). Furthermore, out of 20 *H. pylori* isolates, 17 (85%) were susceptible to >13 non-antimicrobials and 3 (15%) were susceptible to < 13 (*P*-value <0.05). Furthermore, the effective antibacterial concentration of non-antibiotics was much higher than those of antibiotics and also exceeded their plasma concentration. It has been proposed that non-antimicrobial drugs in their low plasma concentration, may act as weak antibacterials, antibiotic adjuvants and immunostimulators. The synergy between antibiotic and adjuvant could effectively reduce the dosing and also negatively affect the emergence of resistance [95]. Overall, it was revealed that the majority of non-antimicrobial drugs used in this study exerted effective antibacterial activity against *H. pylori* isolates. Although *H. pylori* susceptibility rates to non-antimicrobials were similar to those of currently used antibiotics, their mechanism of action remains to be elucidated. Considering the limitation of our study, we can mention the lack of direct *in vivo* studies to confirm the potential antimicrobial effects of drugs’ metabolites or indirect effects via stimulation of the immune system. Furthermore, there is a need for *in vitro* investigating the *anti*-*H. pylori* effect of all the tested non-antibacterials when combined with antibiotics. Because consumption of high concentrations of non-antimicrobials is associated with cytotoxicity and risk of debilitating side effects, combining their sub-inhibitory concentrations with antibiotics would be an important subject for intensive research on these drugs with such a wide range of diversity.

## Funding statement

This research did not receive any specific grant from funding agencies in the public, commercial, or not-for-profit sectors.

## Data availability statement

Data included in article/supplementary material/referenced in article.

## CRediT authorship contribution statement

**Allah Nazar Atif:** Methodology, Investigation. **Atousa Hatefi:** Formal analysis, Data curation. **Asadullah Arven:** Methodology, Investigation. **Alireza Foroumadi:** Resources, Project administration. **Sara Kadkhodaei:** Investigation, Data curation. **Alireza Sadjadi:** Resources, Project administration. **Farideh Siavoshi:** Writing – review & editing, Writing – original draft, Project administration.

## Declaration of competing interest

The authors declare that they have no known competing financial interests or personal relationships that could have appeared to influence the work reported in this paper.
