# Supplementary material for: Consumption of non-antibacterial drugs may have negative impact on Helicobacter pylori colonization in the stomach
Source: Heliyon. 2024 Mar 4;10(5):e27327. doi: 10.1016/j.heliyon.2024.e27327 (PMC10943393; doi:10.1016/j.heliyon.2024.e27327)
Supplement: Multimedia component 1 [file mmc1.docx]

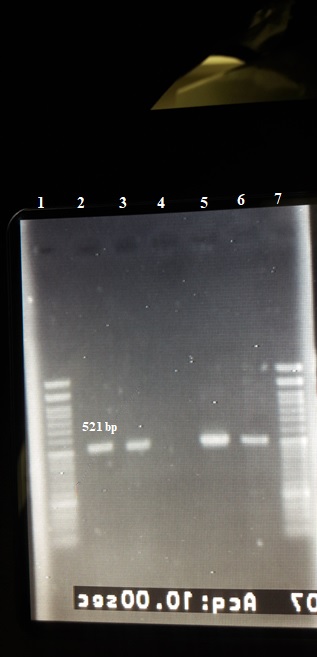


**Full and non-adjusted image of** **Fig. 1. Detection of 16S rDNA in *H. pylori* isolates # 19 and # 20.** Lane 1: Molecular ladder (50 bp), lane 2: 521 bp product from spiral *H. pylori*, lane 3: 521 bp product from mucoid *H. pylori* and lane 4: No template. Lanes 5-7 show repeated electrophoresis of similar PCR products and the ladder: Lane 5: 521 bp product from mucoid *H. pylori*, lane 6: 521 bp product from spiral *H. pylori*, and lane 7: Molecular ladder (50 bp).
